# Exploration of the Association Rules Mining Technique for the Signal Detection of Adverse Drug Events in Spontaneous Reporting Systems

**DOI:** 10.1371/journal.pone.0040561

**Published:** 2012-07-16

**Authors:** Chao Wang, Xiao-Jing Guo, Jin-Fang Xu, Cheng Wu, Ya-Lin Sun, Xiao-Fei Ye, Wei Qian, Xiu-Qiang Ma, Wen-Min Du, Jia He

**Affiliations:** 1 Department of Health Statistics, Second Military Medical University, Shanghai, China; 2 Adverse Drug Reaction Monitoring Centre of Shanghai, Shanghai, China; Sapienza University of Rome, Italy

## Abstract

**Background:**

The detection of signals of adverse drug events (ADEs) has increased because of the use of data mining algorithms in spontaneous reporting systems (SRSs). However, different data mining algorithms have different traits and conditions for application. The objective of our study was to explore the application of association rule (AR) mining in ADE signal detection and to compare its performance with that of other algorithms.

**Methodology/Principal Findings:**

Monte Carlo simulation was applied to generate drug-ADE reports randomly according to the characteristics of SRS datasets. Thousand simulated datasets were mined by AR and other algorithms. On average, 108,337 reports were generated by the Monte Carlo simulation. Based on the predefined criterion that 10% of the drug-ADE combinations were true signals, with RR equaling to 10, 4.9, 1.5, and 1.2, AR detected, on average, 284 suspected associations with a minimum support of 3 and a minimum lift of 1.2. The area under the receiver operating characteristic (ROC) curve of the AR was 0.788, which was equivalent to that shown for other algorithms. Additionally, AR was applied to reports submitted to the Shanghai SRS in 2009. Five hundred seventy combinations were detected using AR from 24,297 SRS reports, and they were compared with recognized ADEs identified by clinical experts and various other sources.

**Conclusions/Significance:**

AR appears to be an effective method for ADE signal detection, both in simulated and real SRS datasets. The limitations of this method exposed in our study, i.e., a non-uniform thresholds setting and redundant rules, require further research.

## Introduction

Pharmacovigilance (PhV) is defined as “the science and activities relating to the detection, assessment, understanding, and prevention of adverse effects or any other drug-related problems” [1]. Because of the limitations of animal studies and clinical trials, the post-marketing safety surveillance of drugs could be crucial for PhV. The major datasets used in the post-marketing safety surveillance of drugs are derived from spontaneous reporting systems (SRSs), which are a proxy for the true population. Nowadays there are a large number of adverse drug events (ADEs) are reported in China. The number reached 638,996 until 2009 and has been increasing yearly. It is challenging for pharmacological experts to efficiently identify associated drug-ADE reports from such large datasets without data mining tools. Therefore, there is an urgent need for effective signal detection methods.

In these regards, automatic filtering data mining methods are considered to provide efficient assistance for the surveillance of drug safety. The proportional reporting ratio (PRR), the adjusted PRR (adjPRR), and the reporting odds ratio (ROR) have been used by the Medicines and Healthcare Products Regulatory Agency (MHRA) and other ADE monitoring centers [2]–[3]. The Bayesian confidence propagation neural network (BCPNN) has been used by the Uppsala Monitoring Centre (UMC) [4]–[5]. Additionally, the United States Food and Drug Administration (FDA) has applied Multi-item Gamma Poisson Shrinker (MGPS) for data mining of large frequency tables [6]–[7]. However, it should be noted that each method has its deficiencies. For example, for the ROR and PRR, the odds ratio and standard error cannot be calculated if the denominator is zero (i.e., specific ADEs did not occur). For the BCPNN, relatively non-transparent Bayesian statistics, issues relating to multiple comparisons, and a lower sensitivity are inevitable [7]–[8]. Most of these algorithms mentioned above used in SRSs are based on disproportional analysis in 2-dimensional contingency tables (Table 1). With these algorithms, the disproportional reports are found as signals, by comparing adverse events of people taking specific drugs with those taking other drugs. However, the accuracies of other disproportional methods for detecting ADE signals depend, to a large extent, on the number of suspected drug reports. If there are few reports, the accuracy of signal detection is greatly reduced [9]. Therefore, we proposed the use of association rule (AR) mining, which searches for frequent item-sets (specific drugs and ADEs that frequently appear at the same time) without being influenced by reports. Before our study, Harpaz et al. [10] employed AR to detect multi-item ADE associations (drugs-ADE, drug-ADEs, and drugs-ADEs) in the FDA’s adverse effect reporting system (AERS), and their results suggested it to be a valid approach. In our study, we compared the ability of AR with that of other recognized methods to detect 2-item associations (drug-ADE) using simulations and demonstrated its application.

**Table 1:** Two-by-two contingency table.

	Suspected ADEs	All other ADEs	Total
Suspected drug	a	b	a + b
All other drugs	c	d	c + d
Total	a + c	b + d	a + b + c + d

Abbreviations: ADE: adverse drug event.

## Methods

### AR Mining

AR mining, put forward by Agrawal et al. in 1993, searches for interesting relationships among items in a given dataset [11]. ARs were expressed in the form of 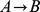
_,_ the 2 items (antecedent A and subsequent B) being from the same dataset, with 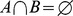. In other words, AR provides information in the form of an “if-then” statement. In the surveillance of ADEs, suspected drug-ADE combinations, such as “
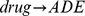
” are evaluated. For example, “thalidomide → deformities” means that if pregnant women take thalidomide, severe birth deformities may occur in their infants.

The strength of an AR is expressed mainly by support, confidence, and lift. The percentage of events that contain A and B among all the events, denoted as 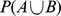, called the support, and this expresses how often the items in a rule are present together. Higher support indicates that the rule, which in this case is the combination of drugs and ADEs, has occurred more frequently and should be detected more easily. The percentage of events including A and B among all events including A, denoted as 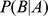, is called the confidence. The confidence provides an estimate for the conditional probability of B given A and is employed to determine the reliability of the rule [12]. Another important parameter in AR, i.e., the lift, is expressed as 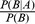. If the lift of the rule is greater than 1, it means the A and B in the same rule are dependent on each other, and the larger the lift, the stronger the association between A and B.

**Table 2:** Ability of AR to detect signals with different Min_lifts.

Min_lift	Sensitivity (%)	Specificity (%)	Youden’s index*
1.0	75.64	79.99	0.536
1.1	71.10	86.29	0.554
1.2	66.86	90.83	0.557
1.3	63.18	93.87	0.551
1.4	60.12	95.87	0.540
1.5	57.74	97.25	0.530
1.6	55.85	98.21	0.521

* Youden’s index  =  (sensitivity + specificity) - 1: the power of finding true positive and true negative signals.

Abbreviations: Min_lift: minimum lift.

**Table 3:** Results of the 5 algorithms on the basis of the simulated datasets.

Algorithms	Generation criteria	Average number ofdetected combinations (SD)	Average number of real signals (SD)
AR	Support ≥3 and lift ≥1.2	284 (17.44)	237 (1.7)
PRR	LI_95_(PRR) >1	199 (11.47)	237 (1.7)
ROR	LI_95_(ROR) >1	198 (11.36)	237 (1.7)
BCPNN	LI_95_(IC*) >0	180 (10.01)	237 (1.7)
MHRA	PRR ≥2 and χ^2^≥4 and a ≥3	132 (5.02)	237 (1.7)

* IC (Information component) is used in the frequency sense in this table but is formulated as a Bayesian metric in the BCPNN.

Based on the 3 parameters above, the procedure for the AR algorithm has 2 steps. Firstly, all the rules with their “support” values being greater than the predefined minimum support (Min_sup) value will remain as frequent item-sets. Secondly, by selecting item-sets whose confidence and lift are both greater than the predefined minimum confidence (Min_conf) and minimum lift (Min_lift) among frequent item-sets generated in the first step, AR accomplishes the procedure of removing parts of the redundant rules. Because we mainly focused on the “drug → ADE” in our study, we then filtered the rules by template matching, choosing the rules in the order of drugs to ADEs by coding the SAS program to ensure that the antecedent of the rules were drug items and the subsequent were ADE items. Pruning rules were also employed to keep only 2-item association rules, when multi-item rules were removed. Finally, suspected 2-item “drug-ADE” association rules were obtained.

**Table 4:** The AUCs of the 3 algorithms.

				**95% Confidence interval**	
**Algorithms**	**Area**	**Standard error**	**P value**	**Lower bound**	**Upper bound**
				0.787	0.789
BCPNN	0.788	0.001	<0.001	0.787	0.790
MHRA	0.759	0.001	<0.001	0.758	0.760

Abbreviations: AUC: area under the ROC curve; BCPNN: the Bayesian confidence propagation neural network; MHRA: the Medicines and Healthcare Products Regulatory Agency; AR: association rule.

**Figure 1:**
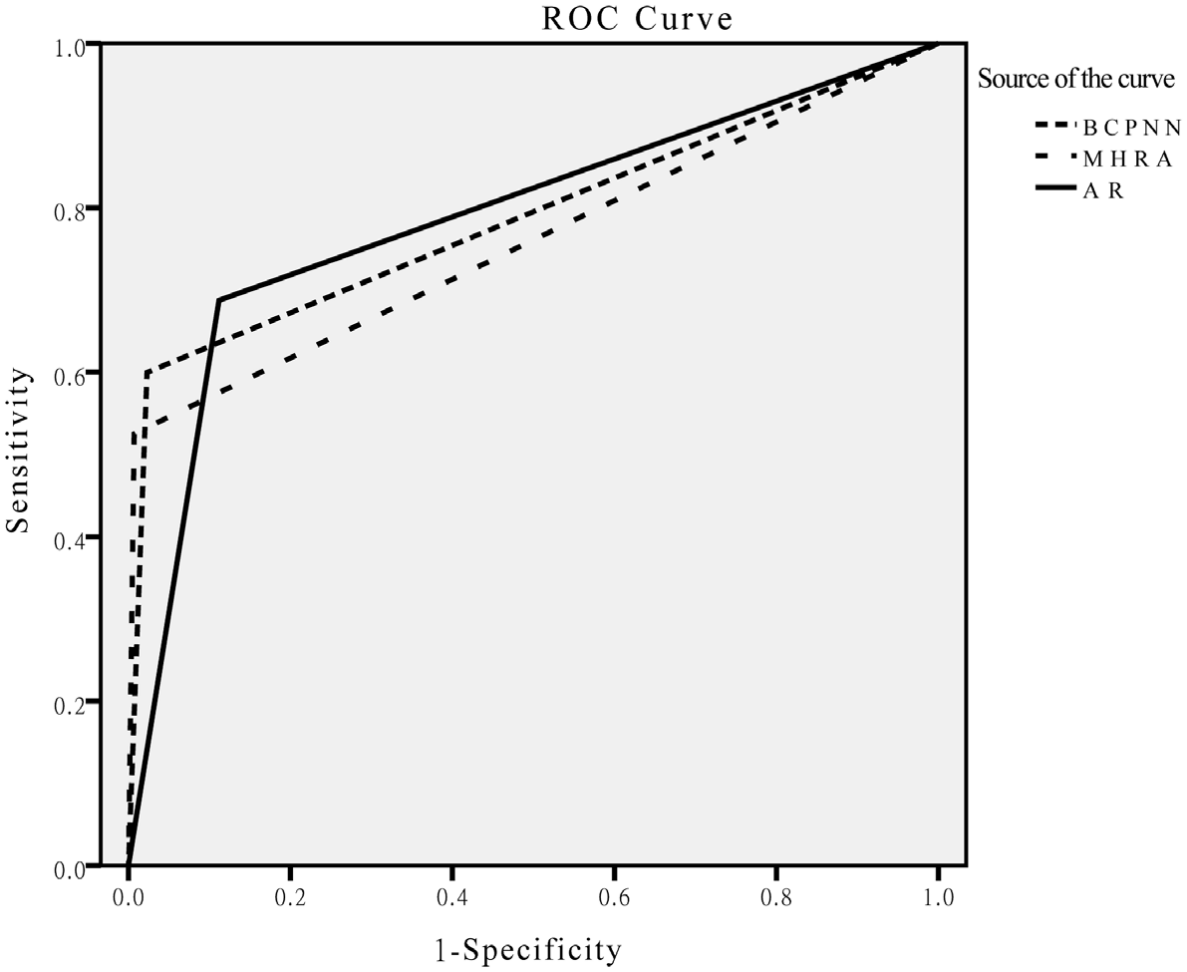
ROC curves for BCPNN, MHRA, and AR. Abbreviations: ROC: Receiver operating characteristic; BCPNN: the Bayesian confidence propagation neural network; MHRA: the Medicines and Healthcare Products Regulatory Agency; AR: association rule.

### Simulation Study [13]–[14]

In this study, we focused on the signal detection of single drug-ADE combinations in simulation data using ROR, PRR, BCPNN, MHRA, and AR, with the BCPNN method being chosen as a representative of the Bayesian methods. Firstly, simulation datasets were generated according to the statistical characteristics of real datasets in SRSs. The SRS model was proposed in the study of Roux [13]. During a given period, the number of reports that are assumed under a Poisson distribution is defined as follows:
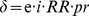
where *e*, *i*, *RR*, and *pr* are the drug exposure frequency, ADE background incidence, relative risk, and reporting probability, respectively. We arbitrarily considered 40 drugs and 60 ADEs (2,400 drug-ADE combinations) to form simulation data. The 2 exposure frequencies of the drugs (*e*) used were 300,000 and 30,000, and each was assigned to half of the drugs. The background incidences (*i*) were 1/200 and 1/500, again each was associated to half of the ADEs. Half of the ADEs were serious and half were mild. In terms of the drug launch duration, we chose 3 different durations: 1, 5, and 10 years, to distribute the dataset. Therefore, the reporting probabilities (*pr*) were defined as 0.095, 0.080, 0.055, 0.030, and 0.010, according to the different drug launch durations and the severities of the ADEs. Only 10% of the drug-ADE combinations were true signals, with RRs of 10, 4.9, 1.5 and 1.2; the remaining 90% were assumed to be coincidental with RRs of 1.

**Table 5:** Ten typical suspected drug-ADE combinations detected by AR in the 2009 Shanghai SRS.

Drug → ADE combinations	Report number	Lift	Bulletin time
Peritoneal dialysate fluid **→** eosinophilia	39	221.24	-
Aspirin **→** gastric ulcer bleeding	16	159.43	-
Cisplatin **→** myelosuppression	14	102.93	-
Simvastatin **→** myalgia	9	71.99	2010-11-17
Estazolam **→** hepatic dysfunction	3	35.06	2010-05-24
Rosiglitazone **→** edema	6	28.84	-
Ossotide **→** phricasmus	4	17.68	2010-03-17
(Bisphosphonates) Zoledronic acid **→** hyperpyrexia	5	5.42	2011-04-15
(Carbostyril) Ciprofloxacin **→** anaphylactoid reaction	14	3.04	2011-01-20
Isotretinoin **→** tetter	3	2.24	2010-08-11

Abbreviations: ADE: adverse drug event.

For the common methods, the reports generated from the simulations were transformed to “a b c d” in a 2-dimensional contingency table for signal detection. AR also required transformation of the data before it could be executed: the frequency “a” of the specific drug-ADE combination needed to be split into signal reports. Before implementing AR to detect suspected signals, it is necessary to establish some criteria to prune redundant rules. According to the MHRA, there should be at least 3 reports for a suspected drug-ADE combination [3], [15], thus we predefined the value of the Min_sup count to be 3. In terms of the Min_lift threshold, different studies have set different values according to their datasets and requirements. Harpaz et al. [10] defined the RR threshold, equivalent to the Min_lift in our study, as 2. Therefore, the Min_lift should at least be greater than 1. In addition, because we only focused on the condition of a single drug causing a single ADE in this study, the items should be 2 in a rule [16]. The “confidence” did not seem to be an appropriate parameter to include for signal detection, since it was possible for rare adverse events to have a low confidence despite being strongly associated with certain drugs [10].

### Application Study

To demonstrate the capability of AR in real-life data sets, we collected reports submitted to the SRS of the Shanghai ADE Monitoring Centre in 2009. Recognized ADEs were identified by clinical/pharmacological experts, the literature, dispensatories and ADE information bulletins from the National Center for ADE Monitoring. Our SRS was similar to WHO Adverse Events Reporting System (AERS). Personal information was not involved in our dataset, and individual identify codes in SRS were marked with random numbers. The study was approved by the Ethics Committee of the Second Military Medical University, Shanghai, China. In our study, all algorithms, as well as the Monte Carlo simulation, were implemented using the software SAS 9.1.3.

## Results

### Simulation Study

Thousand simulation datasets with 60 drugs and 40 ADEs were generated, and the average number of drug-ADE combinations was 108,337 with a standard deviation (SD) of 168. Because of the error associated with the simulation, 237 (SD  = 1.7) signals were produced on average.

At the start, a large number of rules were generated by the AR. Because we mainly focused on the rules in the order of drugs → ADEs, the rules were filtered by template matching to assure that the antecedent of the rules were drug items and the subsequent were ADE items. Predefined signals were considered to be the gold standard by the simulation. By comparing the ability of the signal detection with 7 different Min_lifts, we found when Min_lift was 1.2, with a Min_sup of 3, the Youden’s index was optimum (Table 2). We therefore defined the rules whose lift was greater than 1.2 and support was greater than 3 to be suspected signals. The detection criteria of the ROR, PRR, BCPNN, and MHRA have been applied in many empirical studies [2]–[7]. Table 3 shows the results of 5 algorithms after running 1000 simulated datasets. Compared with the predefined real signals, the average numbers of combinations detected by ROR, PRR, BCPNN, and MHRA were all less than 200, but AR generated 284 suspected combinations.

Moreover, because of ROR, PRR, and MHRA with the same theory, we compared the receiver operating characteristic (ROC) of MHRA and BCPNN with that for AR. The ROC was drawn with combinations that appeared more than or equal to 3 times by SPSS18.0, and the results are presented in Figure 1. As shown in Figure 1 and Table 4, each area under the ROC curve (AUC) is larger than 0.75 (P<0.001), and AR has the same AUC as BCPNN, which is larger than that for MHRA.

### Application Study

The generic names of drugs were standardized and coded according to the catalogue of generic names for common prescription drugs issued by the Ministry of Health of China. ADE names were coded with the WHO adverse drug reaction terminology (WHO-ART) using preferred terms (PT) [17]–[18]. Similarly, according to items  = 2, we focused on combinations of drug-ADE instead of drug-ADE-ADE, drug-drug-ADE, etc. As in our simulation study, the thresholds of Min_sup and Min_lift were set at 3 and 1.2, respectively, and template matching and pruning rules were also employed. On the basis of the advice of clinical experts, the literature, and ADE information bulletins by the National Center for ADE Monitoring, the results of the data mining by AR were evaluated.

After the data was cleaned, there were a total of 24,297 reports from January to December in the 2009 Shanghai SRS dataset and 1512 generic drug names and 805 ADE items were generated by standardization. Insignificant reports were then deleted, for example reports when the drug was only physiological saline or glucose. Finally, a total of 570 suspected drug-ADE associations were generated by AR. By decreasing the lift, we found that 10% of the associations were the most suspected, with lifts greater than 100. Lifts between 10 and 100 were secondary suspected associations, accounting for 29.5% of the associations, and associations with lifts between 1.2 and 10 accounted for 60.5% by template matching. Among the 570 drug-ADE rules, most of the associations had been referred from dispensatories or relational references; the others were infrequent. Table 5 shows 10 typical suspected drug-ADE combinations among the associations detected by AR.

According to the results in Table 5, some well-known examples, such as “aspirin → gastric ulcer bleeding” [19], were selected to check whether they could be detected by AR. The results showed that AR detected the well-established “aspirin → gastric ulcer bleeding” association. There were 16 reports of this combination and the lift value was greater than 100, thus it was considered one of the most suspected associations at the front of mining results. Nevertheless, some suspected combinations, which were unfamiliar to people, were detected, such as “simvastatin → myalgia”. Among the 9 reports of this combination, there were 3 strongly associated with high creatine phosphokinase, and 1 report combined with rhabdomyolysis. All of the reports confirmed that adverse myalgia was related to the administration of simvastatin, as reported previously [20]–[21]. In addition, these associations in table 5 were also supported by the ADE information bulletins from the National Center for ADE Monitoring produced between 2010 and 2011.

## Discussion

AR has already been validated as an effective method for the initial identification of multi-item ADEs in other studies (e.g., Harpaz et al. [10]). However, because there is no gold standard regarding the statistical methods that should be used in order to facilitate accurate ADE signal screening and previous studies had not included any comparison of AR to other methods, we designed a simulation study for comparing AR with other methods. According to the predefined standards, we found that AR efficiently detected suspected drug-ADE combinations with the parameters of lift, support, and confidence. It detected associations among drug-ADE combinations on the basis of item frequencies and its performance was similar to the commonly used methods. In the real SRSs dataset, AR detected both familiar and unfamiliar combinations, including “aspirin → gastric ulcer bleeding” and “simvastatin → myalgia”, most of which appeared with strong associations. These combinations detected by AR were consistent with clinical evidence, case analyses, and ADE bulletins from 2010 to 2011, which validated its use for detecting suspected ADE signals in a timely fashion, thus playing an important role in safety surveillance. ADE signal detection is a process that explores the association between a specific drug and the ADE it may cause. In fact, data mining only generates interesting information regarding the data, and this kind of relationship cannot be considered causal. To be precise, data mining plays a part as a decision support tool, rather than as a decision maker. Therefore, statistical methods based on the execution of computer programs are not a panacea in studying drug safety; large-scale pharmacoepidemiological investigations and pharmacological research are still necessary for the assessment of signals before any definite conclusions can be drawn.

Our study also revealed some challenges that can be attributed to both the method and quality of the data. Support and lift were the main parameters of the AR. Since there were no criteria for setting their threshold values, by virtue of the size of the dataset, subjectivity was the main limitation. In our study, we selected a Min_lift of 1.2 according the largest Youden’s index. Ordonez [16] agreed that a Min_lift of 1.2 would generate primarily predictive rules. Furthermore, we clearly showed (Table 3 and Figure 1) that AR was more sensitive than the other algorithms. Further, there were many false-positives that were difficult to avoid. If we raised the Min_lift and Min_sup to reduce the false-positives, the false-negatives would increase. Because of the absence of recognized settings for the Min_lift and Min_sup, these values should be set rationally according to the following conditions: if detecting as many ADE signals as possible is the primary goal, the Min_lift should be set at higher than but nearer to 1; on the contrary, if eliminating non-association is the primary task, the Min_lift should be set higher. In addition, plenty of redundant rules were inescapable in the process of mining large datasets. The percentages of redundant rules were 84.12% and 87.46% in the simulation and application studies, respectively. Fortunately, a variety of improved methods has been proposed recently [22]–[24]. In our study, template matching and the threshold settings for the support and lift were the main pruning approaches used in signal detection.

Although simulation datasets cannot reflect real-world situations and all conditions exactly, they are still a good alternative to compare the signal detection ability of methods under conditions without a gold standard. It would also be critical for the algorithms to work well in real-world situations. As we can see from our study, the data quality of SRSs seems to be of utmost importance. The authenticity and integrity of datasets tremendously influence the reliability and accuracy of statistical results. In our study, we did a lot of work to improve the quality of the 2009 Shanghai SRS datasets, for example, deleting duplicated reports, standardizing the denominations of the drugs and adverse events, and coding the drugs and adverse events. However, the limitations of SRSs are still inevitable. To mirror the situation more accurately, physicians and nurses should pay closer attention to the details in case of under-reporting [25]. Additionally, the background incidence of the ADE in the whole population and the number of patients exposed to the drug are still unknown, and these are the inherent defects of SRSs [26]. There are a number of factors that can distort the real causal relationship of a drug-ADE association, therefore controlling and assessing these factors are key issues that are worthy of concern at present.

Considering several challenges of SRSs and data mining algorithms, there is still much work to do. In particular, improving AR and other algorithms to assure the accuracy of signals detected in SRSs will be the focus of our future work.
